# PI3K/AKT/SERBP-1 pathway regulates *Alisma orientalis* beverage treatment of atherosclerosis in APOE^−/−^ high-fat diet mice

**DOI:** 10.1080/13880209.2023.2168020

**Published:** 2023-02-24

**Authors:** Ruiyi Liu, Yan Sun, Dong Di, Xiyuan Zhang, Boran Zhu, Haoxin Wu

**Affiliations:** a Key Laboratory of Integrative Biomedicine for Brain Diseases, School of Chinese Medicine, Nanjing University of Chinese Medicine, Nanjing, People's Republic of China; b National Famous Chinese Medicine Expert Inheritance Studio (Meng Jingchun), School of Chinese Medicine, Nanjing University of Chinese Medicine, Nanjing, People's Republic of China; c Laboratory of Febrile Diseases, School of Chinese Medicine, Nanjing University of Chinese Medicine, Nanjing, People's Republic of China; d Sheyang Hospital of Traditional Chinese Medicine, Yancheng, People's Republic of China

**Keywords:** Traditional Chinese medicine, high-fat diet, aortic plaque, lipid metabolism, network pharmacology

## Abstract

**Context:**

Previously, we found *Alisma orientalis* beverage (AOB), a classic traditional Chinese medicine (TCM) formulation, had the potential effect of treating atherosclerosis (AS). The underlying mechanism was still unclear.

**Objective:**

As an extention of our previous work, to investigate the underlying mechanism of action of AOB in the treatment for AS.

**Materials and methods:**

Network pharmacology was conducted using SwissTargetPrediction, GeneCards, DrugBank, Metascape, etc., to construct component-target-pathway networks. *In vivo*, AS models were induced by a high-fat diet (HFD) for 8 consecutive weeks in APOE^−/−^ mice. After the administration of AOB (3.8 g/kg, i.g.) for 8 weeks, we assessed the aortic plaque, four indicators of blood lipids, and expression of the PI3K/AKT/SREBP-1 pathway in liver.

**Results:**

Network pharmacology showed that PI3K/AKT/SREBP-1 played a role in AOB’s treatment for AS (PI3K: degree = 18; AKT: degree = 17). Moreover, we found that the arterial plaque area and four indicators of blood lipids were all significantly reversed by AOB treatment in APOE^−/−^ mice fed with HFD (plaque area reduced by about 37.75%). In addition, phosphorylated expression of PI3K/AKT and expression of SREBP-1 were obviously increased in APOE^−/−^ mice fed with HFD, which were all improved by AOB (PI3K: 51.6%; AKT: 23.6%; SREBP-1: 40.0%).

**Conclusions:**

AOB had therapeutic effects for AS by improving blood lipids and inhibition of the PI3K/AKT/SERBP-1 pathway in the liver. This study provides new ideas for the treatment of AS, as well as new evidence for the clinical application of AOB.

## Introduction

The latest data from the Global Burden of Disease (GBD) showed that approximately 18.6 million people worldwide died of cardiovascular disease in 2019, which has surpassed infectious diseases as the leading cause of death and disability worldwide (Roth et al. 2020). Atherosclerosis (AS) is the main factor leading to the global epidemic of cardiovascular and cerebrovascular diseases, which is mainly characterized by lipid deposition and chronic inflammation in the arterial wall (Roth et al. 2020). Atherosclerosis (AS) is characterized by fibrofatty lesions formed on the inner wall of arteries and is the primary pathological basis of cardiovascular and cerebrovascular diseases (Kobiyama and Ley 2018). Increasing evidence has indicated that hypercholesterolemia-induced vascular inflammation and cholesterol deposition together constitute a risk factor for AS (Koeth et al. 2019). Statins, lipid-lowering drugs such as atorvastatin and rosuvastatin, are the first-line drugs for the treatment of AS in modern medicine and they have significant clinical efficacy in lowering blood lipids, but they can also cause adverse reactions such as liver damage and rhabdomyolysis (Soppert et al. 2020; Aryal et al. 2021). The latest study showed that a serine protease, PCSK9, actively targets LDL-R and causes its excessive accumulation, while PCKS9 inhibitors significantly reduce LDL-C levels and reverse plaque-like changes (Solanki et al. 2018). However, the high cost of the compound and lack of long-term safety and efficacy data limit its use in patients. Therefore, there is an urgent need to find drugs with safe effects and better efficacy.

Hypercholesterolemia is recognized as the main factor leading to AS; reducing blood cholesterol levels is an important way to prevent the development of AS (Francis 2010). Various studies suggested that lipid metabolism mechanisms play a key role in the pathophysiology of AS and that elevated LDL cholesterol leads to AS independent of inflammation, whereas residual cholesterol can drive the inflammatory component of AS (Geovanini and Libby 2018). However, this evidence was not capable of solving the root cause of treating AS. *Alisma orientalis* beverage (AOB) was first recorded in ‘Huangdi Neijing’, an ancient Chinese medical book, consisted of three herbs including *Alismatis rhizoma* (Sam.) Juzep. (Alismataceae) (Zexie), *Atractylodis macrocephalae rhizoma* Koidz. (Asteraceae) (Baizhu), and *Pyrolae calliantha* H. Andres (Pyrolaceae) (Luxiancao) based on the Chinese Pharmacopeia (2020 Edition). A previous study found that AOB can effectively inhibit the progression of atherosclerosis and improvement of blood lipid levels, and its mechanism of mitigating atherosclerosis may be related to gut microbiota and its metabolite (Zhu, Zhai, et al. 2020). However, how AOB influenced blood lipid levels was not identified. Due to the complex components of this formula, it is difficult to explore multiple targets in traditional Chinese medicine (TCM) formulation. Therefore, the underlying mechanism of AOB’s therapeutic actions have not been fully elucidated.

TCM has characteristics of multi-component, multi-target and integrity. Network pharmacology is based on theories of systems biology, genomics, proteomics and other disciplines, using high-throughput omics data analysis, computer simulation and network database retrieval (Hopkins 2008). The technology reveals the network relationship of drug-gene-target-disease interactions, predicts the mechanism of action of drugs through network relationships, evaluates drug efficacy, adverse reactions, etc., explores essential attributes of TCM by referring to the research ideas of network pharmacology, and has achieved good preliminary results in revealing the comprehensive overall effect of multiple pathways, multiple targets and multiple components of TCM (Zeng et al. 2019; Zhang et al. 2019; Zhu, Cai, et al. 2020). This study adopted network pharmacological results to pre-clinical experiments, starting from the material basis of AOB, analyzing and exploring the mechanism of action of AOB in the treatment for AS, and at the same time providing a certain theoretical basis for clinical application and follow-up research.

## Materials and methods

### Collection of chemical components for AOB and screening of active compounds

We used Traditional Chinese Medicine Systems Pharmacology Database and Analysis Platform (TCMSP, https://old.tcmsp-e.com/tcmsp.php) to screen the active ingredients of each herb (Ru et al. 2014). We then identified compounds with oral bioavailability (OB) ≥ 30% (Xu, Zhang, et al. 2012) and drug-likeness (DL) ≥ 0.18 (Jia et al. 2020) in AOB as compounds with pharmacological activity, based on absorption, distribution, metabolism and excretion (ADME) characteristics of the drugs in the body. After the preliminary screening of compounds, the PubChem database (https://pubchem.ncbi.nlm.nih.gov) was used to confirm their molecular structure and name, to improve the credibility of screening results. The identified molecules were entered into the SwissTargetPrediction website (swisstargetprediction.ch) to find the protein targets of the active compounds, and related targets were added based on published literature reports. Then, the screened protein targets were unified in the Uniprot protein database (https://www.uniprot.org) for specification and protein-gene docking for further prediction and analysis.

### Prediction of potential targets of AOB for treatment

GeneCards is a searchable comprehensive database that automatically integrates gene-centric data from approximately 150 web sources, including genomics, transcriptomics, proteomics, genetics, clinical and functional information (Rebhan et al. 1997). With ‘Atherosclerosis’ as the keyword, relevant gene target information was searched in the GeneCards database (https://www.genecards.org) (Rebhan et al. 1997), and potential genes were supplemented using the TTD database (http://db.idrblab.net/ttd/) (Hamosh et al. 2005). When the number of targets is too large, the Score value in the Genecards database can be used for screening. The larger the score value, the closer the relationship between the target and the disease. The median of the Score value is used as the screening value. When there is too much data, multiple screening can be performed to obtain AS-related targets. The intersection of drug component-related targets and AS targets was operated by Venny2.1 (https://bioinfogp.cnb.csic.es/tools/venny/).

### Construction of an active compound-disease-target network

Upload the intersection of targets to the STRING11.0 database (https://string-db.org) (Szklarczyk et al. 2017) to construct a protein-protein interaction (PPI) network model, set the biological species to ‘Homo sapiens’, and set ‘highest confidence’ > 0.9. The PPI network was obtained by screening, and the PPI network was further clustered by Cytoscape_v3.8.2 (Shannon et al. 2003) to obtain potential protein functional modules. The core targets are selected according to the comprehensive ranking of node connectivity (degree), node closeness (closeness) and node betweenness (betweenness).

### Gene Ontology (GO) and Kyoto Encyclopedia of Genes and Genomes (KEGG) enrichment analysis

The core target genes obtained in the above steps were uploaded to the Metascape platform (http://metascape.org) (Zhou et al. 2019), and a threshold of *p* < 0.01 was set. The main biological processes and metabolic pathways were analyzed, including the KEGG pathway, GO biological process, Cell composition, and molecular function enrichment analysis. Then the data were saved and the results were visualized. Finally, a visualization network of ‘prescriptions – traditional Chinese medicine – chemical components – core targets – key pathways’ was built.

### The preparation process of AOB

**Figure 1.**
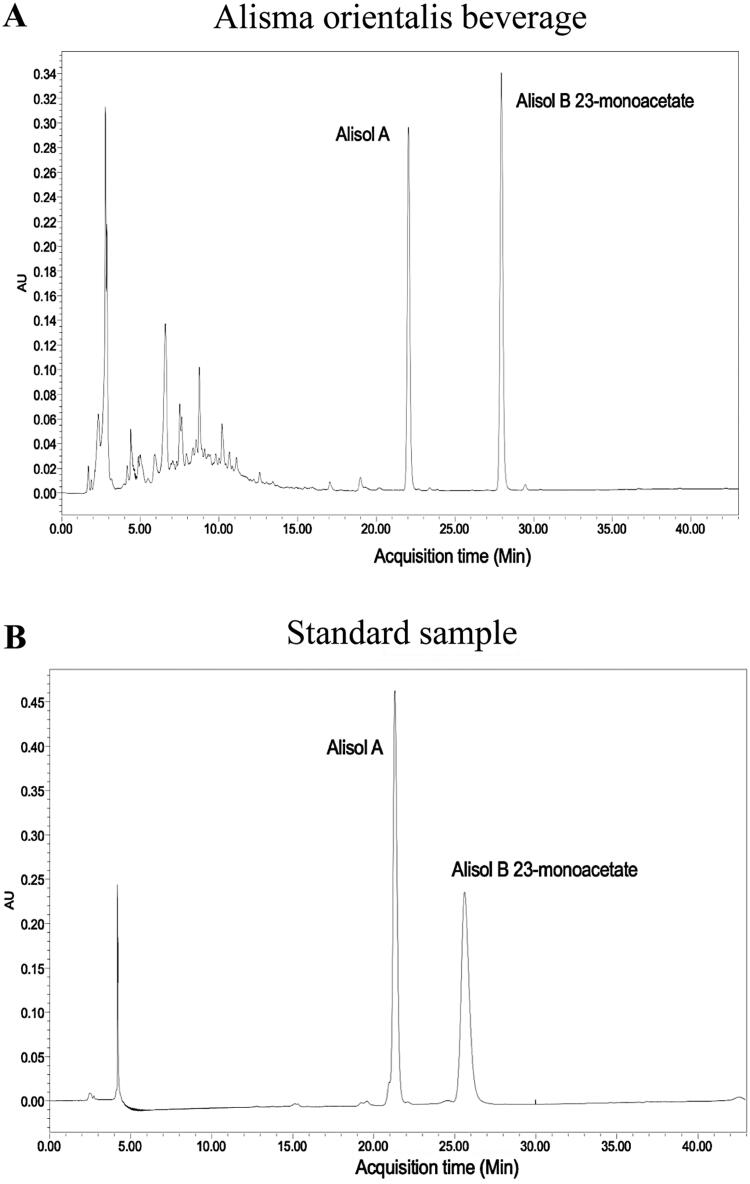
The HPLC fingerprint of AOB (A), benchmark sample (B).

*Alismatis rhizoma*, *Atractylodis macrocephalae rhizoma*, and *Pyrolae herba* were purchased from Tong Ren Tang, Beijing. *Alismatis rhizoma* (100 ± 5 g), *Atractylodis macrocephalae rhizoma* (100 ± 5 g) and *Pyrolae herba* (50 ± 2 g) were placed into 5 L water to soak for 1 h. After soaking, we put the drug at 100 °C for condensation reflux extraction for 2 h and then recycled the medicine liquid and performed rotary evaporation and concentration at a constant temperature of 55 °C. After the concentrated medicinal liquid was recovered, it was freeze-dried at −60 °C and made into freeze-dried powder. The yield is 33.7%, which meets the requirements of drug preparation. The HPLC profile is shown in Figure 1. Both the peak area and concentration of Alisol A and Alisol B 23-monoacetate in AOB are presented in Table 1.

**Table 1.** Components of AOB observed by HPLC.

Both peak area and concentration of Alisol A and Alisol B 23-monoacetate in AOB by HPLC analysis		
Sample	Concentration (mg/mL)	Peak area
Alisol A	0.353	8,586,146
AOB	0.139	3,383,560
Alisol B 23-monoacetate	0.773	8,459,846
AOB	0.396	4,334,478

### Animals

Ten male C57BL/6J mice and 20 male APOE^−/−^ mice were purchased from Changzhou Cavens Laboratory Animal Co., Ltd. (NO. SCXK (SU)-2016-0010). The weight of the mice was 22-24 g and the age of the mice was 4 weeks. All mice were acclimated to a standard rearing environment (Temperature: 18–22 °C, Humidity: 50–60%) for 1 week with 5 mice per cage before experiments carried out. All animal experiments were approved by the Animal Ethics Committee of Nanjing University of Chinese Medicine (NO. 202106A024). All animal experiments complied with animal ethics and all experiments were double-blind.

### High-fat diet model (HFD), experimental design and AOB treatment

We designed three groups including control (CON), model (MOD), *Alisma orientalis* beverage (AOB). In the study, C57BL/6J mice were assigned to the control group, which were fed with a normal mouse diet; and APOE^−/−^ were randomly assigned to the MOD group and AOB group. Mice in the MOD group were fed with a high-fat diet model for 8 weeks; AOB (3.8 g/kg, i.g.) was given daily while feeding with a high-fat diet for 8 weeks in the AOB group. All drugs followed the dosage of the Chinese Pharmacopoeia and did not impair liver and kidney function in mice after 8 weeks of AOB (Table 2).

**Table 2.** Liver function and renal function after AOB treatment for 8 weeks.

	Reference range			
Groups	Liver function		Renal function	
	ALT (10.06–96.47 U/L)	AST (36.31–235.48 U/L)	CR (10.91–85.09 Umol/L)	BUN (10.81–34.74 mg/dL)
CON (Mean ± SD)	43.549 ± 1.655	209.314 ± 4.336	26.314 ± 4.824	22.624 ± 1.106
MOD (Mean ± SD)	74.953 ± 15.255	171.605 ± 8.584	56.165 ± 4.455	15.499 ± 1.232
AOB (Mean ± SD)	59.124 ± 9.392	192.546 ± 9.641	45.406 ± 9.737	13.734 ± 2.006
AOD (Mean ± SD)	68.734 ± 16.558	213.935 ± 18.442	51.105 ± 6.759	14.733 ± 2.396

### Analysis of blood lipids

All mice were anesthetized by using pentobarbital sodium (45 mg/kg; i.p.). After anesthetization, blood was collected by enucleating the eyeball. The collected blood was centrifuged at low speed (3000 rpm) at 4 °C for 10 min, then collected supernatant and stored in −20 °C. High-density lipoprotein cholesterol (HDL-C) and triglycerides (TG), the serum levels of CHO and low-density lipoprotein cholesterol (LDL-C) were measured using a Chemray 240 automatic biochemical analyzer (Wuhan Servicebio Technology, Co., Ltd., China). All experiments were performed as described by the manufacturer.

### Aortic plaque analysis

The heart and the aortic arch were taken out at a low temperature of 4 °C, and the residual blood was washed with 0.01 M PBS. Tissues were placed in a cryostat (Leica, Germany) and serial sections (10 μm) from the aortic sinus to the aortic arch were made from the aortic root according to anatomical markers for histological examination of atherosclerotic aortic sinus lesions. Oil red O staining (ORO) and HE staining were subsequently performed. The plaque area was analyzed using Image Pro Plus 6.0 (Image analysis software, Media Cybernetics, Rockville, MD, USA).

### Western blot

The mouse liver tissue (100 mg/kg) was taken out at a low temperature and placed in a lysate for sufficient grinding to a particle-free state, followed by the addition of protease inhibitors. We then centrifuged for 10 min and took the supernatant for a protein concentration test (protein concentration by BCA method). Moreover, the samples were equalized according to the protein concentration and cooked at 100 °C for 5 min with Loading buffer added until the protein was stable. After the target protein was separated by gel electrophoresis (80 v, 90 min), which was transferred to the PVDF membrane under constant flow (300 mA, 60 min, 4 °C). After 18 h of primary antibody including pPI3K (1:1000), PI3K (1:1000), pAKT (1:1000), AKT (1:1000), SREBP-1 (1:1000), GAPDH (1:5000) incubation at 4 °C, we made 2 h of secondary antibody (IgG-Rabbit, 1:4000) incubation at room temperature (20–26 °C), ECL imaging was performed. Visualization of the blot was performed with the chemiluminescent substrate SuperSignal West Pico (Thermo Fisher Science Inc.) and displayed as density relative to GAPDH. Experiments were performed at least 3 times.

### Statistical analysis

All data were shown in the form of mean ± SEM. One-way ANOVA was used with the honestly important difference from Tukey or the *post-hoc* test from Dunnett. For all statistical tests, GraphPad Prism 8.0 was used, and one-way ANOVA was used in three groups. *p* < 0.05 was considered statistically significant.

## Results

### Identification of potential action targets of AOB

We collected 137 compounds in AOB from the TCMSP database, 46 of which belonged to *Alismatis rhizoma*, 55 belonged to *Atractylodis macrocephalae rhizoma* and 36 belonged to *Pyrolae herba*. After screening by ADME, a total of 7 active ingredients of *Alismatis rhizoma*, 4 from *Atractylodis macrocephalae rhizoma*, 5 from *Pyrolae herba*, and 1 common active ingredient from *Alismatis rhizoma* and *Pyrolae herba* were obtained, including Alisma alcohol, kaempferol, quercetin, gallic acid, atractylodes lactone, etc. (Table 3). Furthermore, we collected the targets of 17 active compounds in AOB from the TCMSP. After the integration of UniProt database entries and the deletion of duplicates, 601 targets were obtained (Table 4).

**Table 3.** The active compounds of AOB.

*Alisma orientalis* beverage				
Mol ID	Herb name	Molecule name	OB (%)	DL
MOL000359	*Alisma orientale* (Sam.) Juz. *Pyrola calliantha* H. Andres	β-Sitosterol	36.91	0.75
MOL000831	*Alisma orientale* (Sam.) Juz.	Alisol B monoacetate	35.58	0.81
MOL000832	*Alisma orientale* (Sam.) Juz.	Alisol B 23-acetate	32.52	0.82
MOL000849	*Alisma orientale* (Sam.) Juz.	16β-Methoxyalisol B monoacetate	32.43	0.77
MOL000853	*Alisma orientale* (Sam.) Juz.	Alisol B	36.76	0.82
MOL000854	*Alisma orientale* (Sam.) Juz.	Alisol C	32.7	0.82
MOL000856	*Alisma orientale* (Sam.) Juz.	Alisol C monoacetate	33.06	0.83
MOL002464	*Alisma orientale* (Sam.) Juz.	1-Monolinolein	37.18	0.3
MOL000033	*Atractylodes macrocephala* Koidz.	(24*S*)-24-Propylcholesta-5-ene-3beta-ol	36.23	0.78
MOL000028	*Atractylodes macrocephala* Koidz.	α-Amyrin	39.51	0.76
MOL000049	*Atractylodes macrocephala* Koidz.	3β-Acetoxyatractylone	54.07	0.22
MOL000072	*Atractylodes macrocephala* Koidz.	8β-Ethoxy atractylenolide III	35.95	0.21
MOL000422	*Pyrola calliantha* H. Andres	Kaempferol	41.88	0.24
MOL000552	*Pyrola calliantha* H. Andres	5,2′-Dihydroxy-6,7,8-trimethoxyflavone	31.71	0.35
MOL000553	*Pyrola calliantha* H. Andres	(−)-Chimonanthine	38	0.66
MOL000569	*Pyrola calliantha* H. Andres	Digallate	61.85	0.26
MOL000098	*Pyrola calliantha* H. Andres	Quercetin	46.43	0.28

**Table 4.** The targets of active compounds in AOB.

Targets of AOB							
NPC1L1	HTR2A	PLA2G2A	IL2	IDH1	ABCC1	CCR1	HSD17B1
NR1H3	HTR2C	CSNK2A1	UCK2	PDE3A	AHR	CNR1	TYMS
RORC	MMP1	GSK3B	NCS1	FNTA	ABCB1	C5AR1	AKR1C1
HMGCR	MAPK14	TTPA	DPEP1	NAMPT	CYP1B1	DUT	CHIT1
SHBG	PGA5	FABP6	HCK	GPR88	ABCG2	JAK3	CTSB
CYP51A1	FNTB	PDK2	IMPDH2	HSD17B2	ADORA1	KDR	SRC
CYP17A1	PGGT1B	PPARA	YARS1	FKBP1A	CA4	DRD3	GSR
CYP19A1	OXTR	ADK	CTSF	PDE2A	ALOX15	MDM2	AURKA
SREBF2	IKBKB	DHFR	CSK	PYGM	ALOX12	AVPR1A	CSNK1G2
AR	TTR	SYK	RARB	MAST3	PTPRS	PDE10A	NOS3
RORA	BMP2	FGFR1	AMY2A	CETP	ADORA2A	EDNRB	SORD
ESR1	CFB	EPHX2	RNASE3	CCNB3	CCNB3	MC4R	LGALS7
ESR2	MAPK10	CTNNA1	AMY1A	CASR	GPR35	MC1R	LGALS7B
PTPN1	THRB	TRAPPC3	AMY1B	GABRB3	DAPK1	HSD17B3	DPP4
CYP2C19	ALB	ELANE	AMY1C	PSENEN	MPG	TK2	ADAM17
SLC6A2	PGR	CTSK	BHMT	NCSTN	SLC22A12	MC5R	APCS
ACHE	CA2	ITK	ABL1	APH1A	TNKS2	MC3R	ADH1B
SERPINA6	MAPKAPK2	PCK1	B3GAT1	PSEN1	TNKS	AVPR2	HSP90AA1
G6PD	EPHB4	MET	MMP9	APH1B	MPO	CNR2	F10
BCHE	ITGAL	GSTA1	RXRB	GABRA3	PTK2	MAP3K14	PNPO
SLC6A4	PIM1	FABP7	ACE	GABRG2	CA3	ADA	CFD
CHRM2	PPIA	PAH	RAB11A	CDK1	CA6	DRD4	ANG
NR1I3	CASP7	PDE3B	C1S	CCNB1	PKN1	NR3C2	SEC14L2
NR1H2	ANXA5	PRKACA	DTYMK	CCNB2	CA14	NR3C1	HSPA8
DHCR7	MAOB	AMD1	CD1A	PDGFRB	NEK2	CCKBR	FABP4
PTGER1	AKR1C2	MMP12	JAK2	ROCK2	CXCR1	CHUK	CTSV
PTGER2	MAPK8	SOD2	GPI	LIMK2	CAMK2B	KCNA5	LCK
VDR	TREM1	FGFR2	ACADM	CDK9	AKT1	SLC6A3	C1R
TBXAS1	GSTP1	MMP8	GCK	IRAK4	NEK6	SMO	MTHFD1
PTGES	MMP3	PPP5C	EPHA2	LRRK2	CA5A	FAAH	IGLV2-8
PPARD	AKR1B1	CYP2C9	AKT2	AURKAIP1	AXL	MAPK1	NQO1
CES2	CES1	ADH5	GRB2	NPY5R	NUAK1	F2RL1	PARP1
HSD11B1	GC	CTSG	TEK	TK1	AKR1C4	REN	ESRRG
SQLE	HSD17B11	ALDH2	SETD7	AMPD2	CA13	JAK1	HDAC8
PTPN6	KIF11	CDA	PAPSS1	FASN	AKR1A1	MCHR1	PLA2G10
PTPN2	CHEK1	AZGP1	ALAD	TRPV1	APP	F2	ADH1C
GLRA1	PDE4B	PLAU	GSTT2B	S1PR3	CD38	STS	RXRA
NOS2	GBA	CBR1	TGFB2	S1PR1	TOP1	ALK	PLK1
PPARG	PIK3CG	ATOX1	LGALS3	ALOX5	CFTR	CSF1R	BCAT2
UGT2B7	PNP	CA1	NMNAT1	LPAR6	GRK6	DRD1	PDPK1
POLB	MMP13	FHIT	RAC2	LPAR5	CCNB1	SCD	HTR3A
PRKCA	FKBP1A	RBP4	LTA4H	ENPP2	ODC1	ACP1	NOS1
PRKCD	APOA2	FECH	AGXT	SLC8A1	ADORA3	AKR1B10	ADRA2A
PRKCQ	CMA1	SHMT1	PAK6	CTSL	MCL1	CD81	ADRA2C
PTGS2	CCNA2	ISG20	PIK3R1	PREP	PLG	ARG1	HRH2
FNTA	CASP3	BLVRB	CLK1	S1PR5	PLA2G7	FOLH1	ADRA2B
VAV1	PDE5A	AHCY	RHOA	S1PR4	MAPT	GSTM1	CXCR4
PGGT1B	DHODH	FABP3	MAN1B1	ACACB	TOP2A	NT5M	SCARB1
F2R	CDK5R1	SULT1E1	PPP1CC	PIK3CD	APEX1	TPSB2	SCN4A
TACR1	CDK2	SULT2B1	ARSA	PIM3	GUSB	FKBP3	CXCR3
PRKCB	MIF	DCK	MME	PFKFB3	HSP90AB1	INSR	SIGMAR1
PDE4D	TNNC1	FABP5	PLEKHA4	AURKB	RET	WARS1	PRCP
METAP2	TGFBR1	ABO	F11	TYK2	NAE1	RFK	ADRA1D
P2RX3	SULT2A1	REG1A	IMPDH1	F9	PCSK7	PROCR	CTSC
TRPV4	EGFR	NR1I2	HRAS	KCNH2	DRD5	FGG	KHK
PRKCG	PTPN11	ERBB4	PCTP	ERBB2	HRH4	MAOA	ARHGDIA
PRKCE	NR1H4	ME2	TPH1	PTK6	HTR1E	PLA2G1B	SSTR4
PRKCH	WAS	XIAP	HAGH	FDFT1	HTR5A	RPS6KA5	SSTR1
BACE2	BACE1	F7	BCL2L1	TERT	OPRL1	GLRA2	CACNA1G
CTSD	AKR1C3	KYAT1	CASP1	FABP1	OPRK1	ATP12A	PIM2
PER2	GLO1	HNF4G	THRA	ZAP70	MMP2	PYGL	HNMT
MTAP	IGF1R	RARG	ESRRA	CDK6	SDS	IGF1	NQO2
MAP2K1	PSAP	GM2A	MMP7	S100A9	CYP2C8	CTSS	BST1
HK1	RARA	LCN2	PNMT	PGF	SERPINA1	ACP3	ALDOA
HPN	CRABP2	TGM3	CCNT1	CCL5	C8G	BIRC7	PADI4
TPI1	TTL	CDK5R1	CACNA1B	PSEN2	CA12	CA9	CDC25A
CDC25B	RIPK3	RIPK2	CDK5	PSENEN	NCSTN	APH1A	PSEN1
NOX4	XDH	TYR	FLT3	CA7	HEXB	FDPS	GNPDA1
STAT1	LGALS2	CDK7	KIT	GSTO1	PITPNA	EIF4E	MTNR1A
MTNR1B	ACKR3	HSD11B2	SCN9A	PSEN2	OPRM1	OPRD1	CCNC
CDK8	IL6ST	MTOR	PIK3CA	PDGFRB	NTRK1	PAK1	PIK3CB
HCRTR2	HCRTR1	KCNK3	GYS1	POLA1	FYN	PDGFRA	EPHB3
GSK3A	VHL	EZH2	FAP	P2RX7	CHRNB3	HASPIN	KCNN4
SSTR3	MAP3K8	NOD1	NOD2	TRPM8	PHLPP2	SLC6A1	DRD2
CCNA1	CHRNA6	CHRNB2	CHRNA3	SERPINE1	TUBB1	FUT7	KDM4E
MYLK							

**Figure 2.**
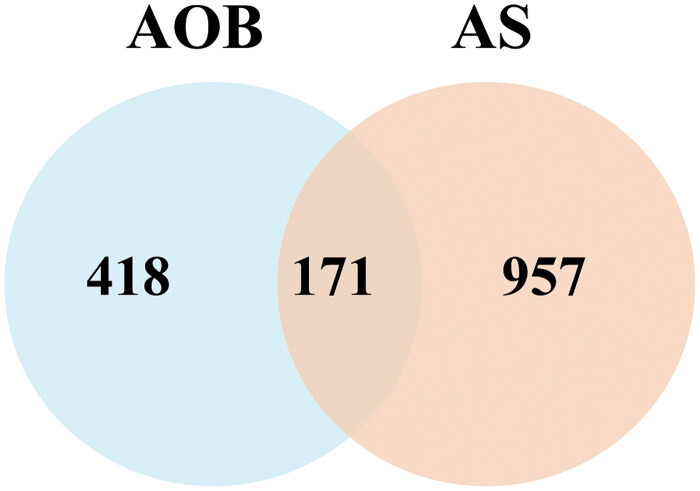
Targets screening involved in AOB for the treatment of AS. Venn diagram of disease targets.

We collected 4481 AS targets from the Genecards database. The median of the Score value was used as the screening value, so the target with a Score > 2.77 was set as the potential target of AS. We combined with OMIM, TTD, and DRUGBANK databases to supplement relevant targets, and deleted duplicate values after merging, and finally obtaines 1128 dyslipidemia-related targets. We took the intersection of the screened drug active ingredient targets and AS targets, and drew Venn diagrams through Venny2.1 to obtain 171 common targets of AOB and AS (Figure 2 and Table 5).

**Table 5.** The 171 common targets of AOB and AS.

Common targets of AOB and AS							
NPC1L1	PTGS2	JAK1	GC	NOS3	SOD2	CSK	BCL2L1
NR1H3	PGGT1B	F2	GBA	DPP4	MMP8	BHMT	CASP1
HMGCR	F2R	CSF1R	PIK3CG	ADAM17	CYP2C9	MMP9	STAT1
SHBG	PDE4D	HTR2A	MMP13	HSP90AA1	ALDH2	ACE	LGALS2
CYP19A1	PRKCE	MMP1	APOA2	F10	AHCY	C1S	GSTO1
SREBF2	CTSD	MAPK14	CMA1	FABP4	FABP3	JAK2	PITPNA
AR	CCR1	IKBKB	CASP3	NQO1	REG1A	AKT2	PSEN2
ESR1	CNR1	TTR	PDE5A	PARP1	NR1I2	TEK	IL6ST
ESR2	C5AR1	MAPK10	MIF	PLA2G2A	F7	TGFB2	MTOR
CYP2C19	KDR	ALB	TGFBR1	GSK3B	MMP2	LGALS3	PIK3CA
ACHE	EDNRB	ITGAL	EGFR	TTPA	IGF1	LTA4H	PIK3CB
BCHE	NR3C2	ANXA5	PTPN11	PPARA	MMP7	AGXT	NAMPT
SLC6A4	NR3C1	MAOB	NR1H4	SYK	S100A9	PIK3R1	CETP
NR1H2	CHUK	MAPK8	BACE1	EPHX2	LCN2	RHOA	CASR
PPARD	SLC6A3	GSTP1	CHIT1	CTNNA1	PGF	ARSA	PSEN1
NOS2	MAPK1	MMP3	SRC	ELANE	CCL5	MME	LRRK2
PPARG	REN	CES1	GSR	MMP12	IL2	F11	ALOX5
POLB	AKT1	APP	TOP1	CFTR	PLG	PLA2G7	MAPT
HTR3A	NOS1	CXCR4	SCARB1	CXCR3	KHK	DRD2	SERPINE1
CTSL	F9	KCNH2	FDFT1	ARG1	GSTM1	PROCR	FGG
PLA2G1B	NOX4	XDH	ABCC1	ABCB1	ALOX15	ADORA2A	MPO
PTK2	APEX1	MYLK					

### The potential targets of AOB for the treatment of AS

**Figure 3.**
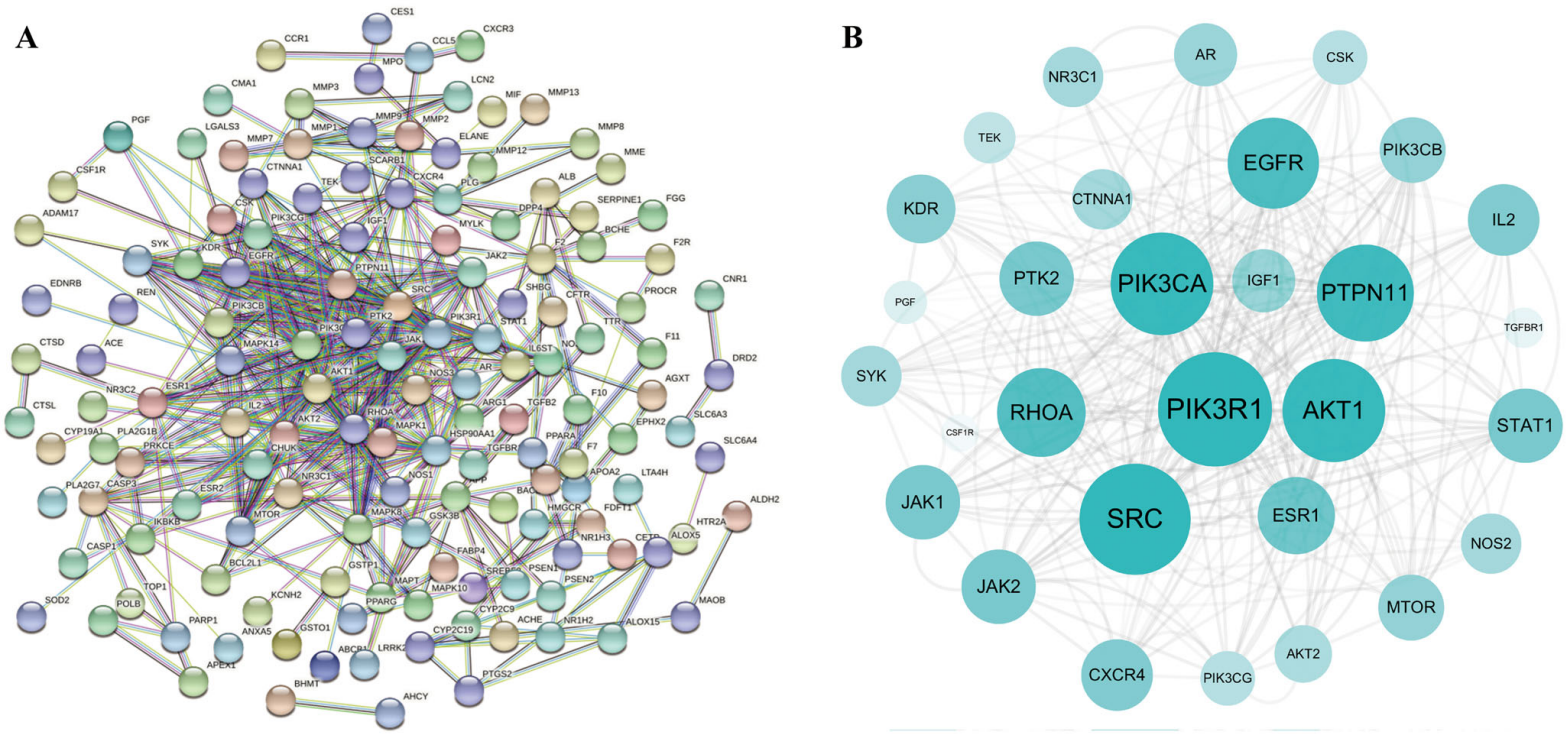
The potential targets of AOB for the treatment of AS. (A) The PPI network was constructed by the STRING database. (B) Drawing the PPI core network with Cytoscape 3.8.2 for visual display.

To comprehensively elucidate the possible mechanism of AOB in the treatment of AS, 171 AOB anti-AS target gene names were imported into the STRING database to construct a PPI network. The required interaction score was 0.9 and the disconnected node network was hidden to draw a PPI network graph (Figure 3(A)). To achieve better visualization and identify core targets, we build a network using Cytoscape based on target degrees. With this network, core targets were obtained: PIK3R1, AKT1, PIK3CA, MAPK1, PTPN11, EGFR and MAPK4 (Figure 3(B) and Table 6). These targets may be considered as primary targets of action for AOB for AS treatment, and their identification suggests that AOB treats AS through multiple potential targets.

**Table 6.** The top 10 targets of the PPI Network.

Target	Betweenness centrality	Closeness centrality	Degree
HSP90AA1	0.166047284	0.432343234	60
PIK3R1	0.081179043	0.411949686	58
SRC	0.133317904	0.433774834	56
AKT1	0.074995392	0.401840491	50
PIK3CA	0.029786286	0.384164223	50
MAPK1	0.109227209	0.413249211	48
PTPN11	0.023755181	0.388724036	46
EGFR	0.032745575	0.388724036	42
RHOA	0.058774436	0.389880952	40
MAPK14	0.039497188	0.374285714	32

### GO and KEGG enrichment analysis for identification of the pathway mechanisms of AOB

**Figure 4.**
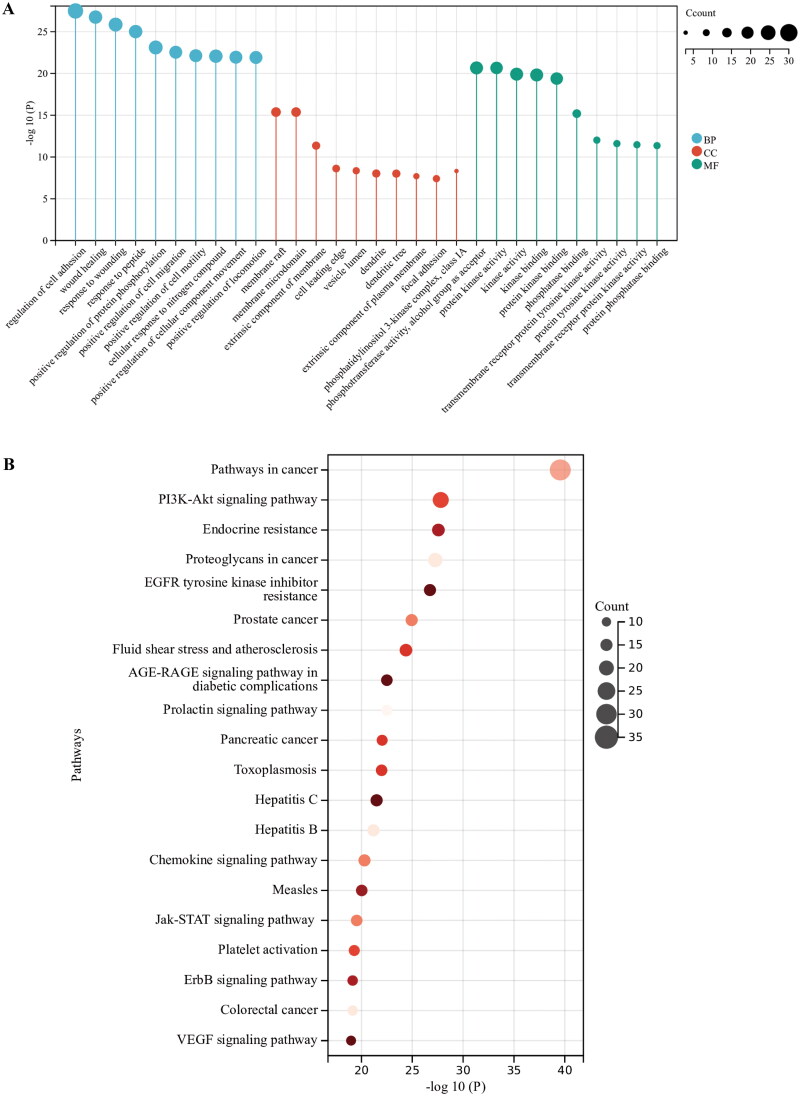
GO and KEGG enrichment analysis for identification of the pathway mechanisms of AOB. (A) The top 10 significantly enriched (*p* < 0.01) terms in BP, CC and MF of GO analysis were selected. (B) The top 20 pathways with significant enrichment (*p* < 0.01) were selected.

The Metascape data platform was used to analyze the signal pathway of the related targets in the regulation of AS by AOB. AOB was mainly involved in the biological processes including regulation of cell adhesion, wound healing, positive regulation of protein phosphorylation, positive regulation of cell migration, etc. The main cellular components involved include membrane raft, the extrinsic component of the membrane, phosphatidylinositol 3-kinase complex, focal adhesion, etc. GO molecular functions of AOB involved include phosphotransferase activity, alcohol group as acceptor, protein kinase activity, kinase activity, kinase binding, protein kinase binding, phosphatase binding, transmembrane receptor protein tyrosine kinase activity, protein tyrosine kinase activity, transmembrane receptor protein kinase activity, protein phosphatase binding. The pathways involved mainly include cancer pathways, PI3K-AKT signaling pathway, EGFR tyrosine kinase inhibitor resistance, fluid shear stress and atherosclerosis, AGE-RAGE signaling pathway in diabetic complications, hepatitis B, etc. (Table 7). The top 10 significantly enriched (*p* < 0.01) terms in BP, CC and MF of GO analysis were selected (Figure 4(A)). The top 20 pathways with significant enrichment (*p* < 0.01) were selected (Figure 4(B)).

**Table 7.** KEGG enrichment analysis results.

Category	GO	Description	LogP	Count
KEGG pathway	hsa05200	Pathways in cancer	−39.55364346	31
KEGG pathway	hsa04151	PI3K-Akt signaling pathway	−27.80028128	22
KEGG pathway	hsa01522	Endocrine resistance	−27.56991838	16
KEGG pathway	hsa05205	Proteoglycans in cancer	−27.25628276	19
KEGG pathway	hsa01521	EGFR tyrosine kinase inhibitor resistance	−26.73995834	15
KEGG pathway	hsa05215	Prostate cancer	−24.94056688	15
KEGG pathway	hsa05418	Fluid shear stress and atherosclerosis	−24.3778864	16
KEGG pathway	hsa04933	AGE-RAGE signaling pathway in diabetic complications	−22.50156695	14
KEGG pathway	hsa04917	Prolactin signaling pathway	−22.49866763	13
KEGG pathway	hsa05212	Pancreatic cancer	−22.03626506	13
KEGG pathway	hsa05160	Hepatitis C	−21.49278225	15
KEGG pathway	hsa05161	Hepatitis B	−21.18256658	15
KEGG pathway	hsa04062	Chemokine signaling pathway	−20.30080047	15
KEGG pathway	hsa05162	Measles	−20.02945253	14
KEGG pathway	hsa04630	Jak-STAT signaling pathway	−19.53523346	14
KEGG pathway	hsa04611	Platelet activation	−19.29312092	13
KEGG pathway	hsa04012	ErbB signaling pathway	−19.13592737	12
KEGG pathway	hsa05210	Colorectal cancer	−19.13592737	12
KEGG pathway	hsa04370	VEGF signaling pathway	−18.98104023	11
KEGG pathway	hsa04014	Ras signaling pathway	−18.65825684	15
KEGG pathway	hsa04915	Estrogen signaling pathway	−18.65571774	13
KEGG pathway	hsa04931	insulin resistance	−18.13117073	12
KEGG pathway	hsa04510	Focal adhesion	−18.11591861	14
KEGG pathway	hsa04668	TNF signaling pathway	−18.0360695	12
KEGG pathway	hsa04072	Phospholipase D signaling pathway	−17.9771753	13
KEGG pathway	hsa04066	HIF-1 signaling pathway	−17.85103726	12
KEGG pathway	hsa05164	Influenza A	−17.34180853	13
KEGG pathway	hsa04380	Osteoclast differentiation	−17.0538665	12
KEGG pathway	hsa04550	Signaling pathways regulating pluripotency of stem cells	−16.50259738	12
KEGG pathway	hsa05142	Chagas disease (American trypanosomiasis)	−16.46447588	11
KEGG pathway	hsa04660	T cell receptor signaling pathway	−16.19441652	11
KEGG pathway	hsa04015	Rap1 signaling pathway	−16.05447112	13
KEGG pathway	hsa04664	Fc epsilon RI signaling pathway	−15.92581654	10
KEGG pathway	hsa04670	Leukocyte transendothelial migration	−15.8570988	11
KEGG pathway	hsa04919	thyroid hormone signaling pathway	−15.77645797	11
KEGG pathway	hsa04722	Neurotrophin signaling pathway	−15.73665629	11
KEGG pathway	hsa04071	Sphingolipid signaling pathway	−15.61925748	11
KEGG pathway	hsa04068	foxo signaling pathway	−15.24808488	11
KEGG pathway	hsa05222	Small cell lung cancer	−14.90283077	10
KEGG pathway	hsa04210	Apoptosis	−14.77385153	11
KEGG pathway	hsa04914	Progesterone-mediated oocyte maturation	−14.72482596	10
KEGG pathway	hsa05213	Endometrial cancer	−14.6007413	9
KEGG pathway	hsa04150	mTOR signaling pathway	−14.55382048	11
KEGG pathway	hsa05169	Epstein-Barr virus infection	−14.52462989	13
KEGG pathway	hsa05224	Breast cancer	−14.43264667	11
KEGG pathway	hsa0460	Toll-like receptor signaling pathway	−14.38982393	10
KEGG pathway	hsa05214	Glioma	−13.97694069	9
KEGG pathway	hsa04662	B cell receptor signaling pathway	−13.8230983	9
KEGG pathway	hsa05220	Chronic myeloid leukemia	−13.77316427	9
KEGG pathway	hsa05231	Choline metabolism in cancer	−12.69949295	9
KEGG pathway	hsa05221	Acute myeloid leukemia	−12.42231383	8
KEGG pathway	hsa01524	Platinum drug resistance	−12.27052195	8
KEGG pathway	hsa05166	HTLV-I infection	−12.24154346	11
KEGG pathway	hsa05230	Central carbon metabolism in cancer	−12.03171822	8
KEGG pathway	hsa04360	Axon guidance	−11.88732319	10
KEGG pathway	hsa04140	Regulation of autophagy	−11.8636379	9
KEGG pathway	hsa05218	Melanoma	−11.80864445	8
KEGG pathway	hsa05203	Viral carcinogenesis	−11.56800581	10
KEGG pathway	hsa04910	Insulin signaling pathway	−11.2303346	9
KEGG pathway	hsa04930	Type II diabetes mellitus	−11.08590908	7
KEGG pathway	hsa04810	Regulation of actin cytoskeleton	−11.08538891	10
KEGG pathway	hsa04750	inflammatory mediator regulation of trp channels	−11.00553138	8
KEGG pathway	hsa04666	Fc gamma R-mediated phagocytosis	−10.93815983	8
KEGG pathway	hsa04932	Non-alcoholic fatty liver disease (NAFLD)	−10.89148672	9
KEGG pathway	hsa04213	Longevity regulating pathway – multiple species	−10.87448477	7
KEGG pathway	hsa04725	Cholinergic synapse	−10.65068367	8
KEGG pathway	hsa05223	Non-small cell lung cancer	−10.49206217	7
KEGG pathway	hsa05211	Renal cell carcinoma	−9.997610762	7
KEGG pathway	hsa05100	Bacterial invasion of epithelial cells	−9.88593232	7
KEGG pathway	hsa04024	cAMP signaling pathway	−9.763259543	9
KEGG pathway	hsa04211	Longevity regulating pathway	−9.118717848	7
KEGG pathway	hsa04152	AMPK signaling pathway	−8.60443639	7
KEGG pathway	hsa04650	Natural killer cell mediated cytotoxicity	−8.18812509	7
KEGG pathway	hsa04960	Aldosterone-regulated sodium reabsorption	−8.16782521	5
KEGG pathway	hsa05146	Amoebiasis	−7.517592689	6
KEGG pathway	hsa04973	Carbohydrate digestion and absorption	−7.478108164	5
KEGG pathway	hsa04923	Regulation of lipolysis in adipocytes	−7.277689618	5
KEGG pathway	hsa00562	Inositol phosphate metabolism	−3.494032868	3
KEGG pathway	hsa04070	Phosphatidylinositol signaling system	−3.124304392	3
KEGG pathway	hsa05145	Toxoplasmosis	−21.98392135	14
KEGG pathway	hsa05152	Tuberculosis	−17.03489964	13
KEGG pathway	hsa04659	Th17 cell differentiation	−16.23835335	11
KEGG pathway	hsa04658	Th1 and Th2 cell differentiation	−11.21614661	8
KEGG pathway	hsa04621	NOD-like receptor signaling pathway	−10.51759803	9
KEGG pathway	hsa05140	Leishmania infection	−8.5243026	6
KEGG pathway	hsa05168	Herpes simplex infection	−7.518063175	7
KEGG pathway	hsa04657	IL-17 signaling pathway	−15.0886455	10
KEGG pathway	hsa04010	MAPK signaling pathway	−12.6446435	12
KEGG pathway	hsa05120	Epithelial cell signaling in Helicobacter pylori infection	−12.37095867	8
KEGG pathway	hsa05133	Pertussis	−8.248605395	6
KEGG pathway	hsa05131	Shigellosis	−6.926342175	5
KEGG pathway	hsa04728	Dopaminergic synapse	−5.489969573	5
KEGG pathway	hsa05132	Salmonella infection	−4.656866744	4
KEGG pathway	hsa04622	RIG-I-like receptor signaling pathway	−3.580079392	3
KEGG pathway	hsa04723	Retrograde endocannabinoid signaling	−2.523302304	3
KEGG pathway	hsa04920	Adipocytokine signaling pathway	−12.3203688	8
KEGG pathway	hsa04922	Glucagon signaling pathway	−3.076284659	3
KEGG pathway	hsa05219	Bladder cancer	−9.890157853	6
KEGG pathway	hsa04912	GnRH signaling pathway	−7.720652493	6
KEGG pathway	hsa04520	Adherens junction	−6.543089003	5
KEGG pathway	hsa04921	Oxytocin signaling pathway	−6.462524366	6
KEGG pathway	hsa04540	Gap junction	−3.295118179	3
KEGG pathway	hsa04371	Apelin signaling pathway	−9.815534753	8
KEGG pathway	hsa04022	cGMP-PKG signaling pathway	−7.718646373	7
KEGG pathway	hsa04261	Adrenergic signaling in cardiomyocytes	−5.261253948	5
KEGG pathway	hsa05206	MicroRNAs in cancer	−7.279201986	8
KEGG pathway	hsa04270	Vascular smooth muscle contraction	−2.767307962	3
KEGG pathway	hsa04726	Serotonergic synapse	−7.241068175	6
KEGG pathway	hsa00590	Arachidonic acid metabolism	−3.751970004	3
KEGG pathway	hsa05010	Alzheimer’s disease	−4.942122898	5
KEGG pathway	hsa05202	Transcriptional misregulation in cancer	−4.573319263	5
KEGG pathway	hsa04064	NF-kappa B signaling pathway	−4.45368227	4
KEGG pathway	hsa04215	Apoptosis – multiple species	−4.598201729	3
KEGG pathway	hsa05014	Amyotrophic lateral sclerosis (ALS)	−3.881447695	3
KEGG pathway	hsa04137	Mitophagy – animal	−3.67260526	3
KEGG pathway	hsa04115	p53 signaling pathway	−3.461237887	3
KEGG pathway	hsa04144	Endocytosis	−3.988916403	5
KEGG pathway	hsa04060	Cytokine-cytokine receptor interaction	−3.603564725	5
KEGG pathway	hsa00330	Arginine and proline metabolism	−3.731646229	3
KEGG pathway	hsa04020	Calcium signaling pathway	−3.366224834	4
KEGG pathway	hsa04530	Tight junction	−3.589422319	4
KEGG pathway	hsa04310	Wnt signaling pathway	−2.50874465	3
KEGG pathway	hsa05323	Rheumatoid arthritis	−3.17432923	3
KEGG pathway	hsa04114	Oocyte meiosis	−2.832488553	3

### Network-based revelation of Compound-Disease-Pathway-Target network correlations

**Figure 5.**
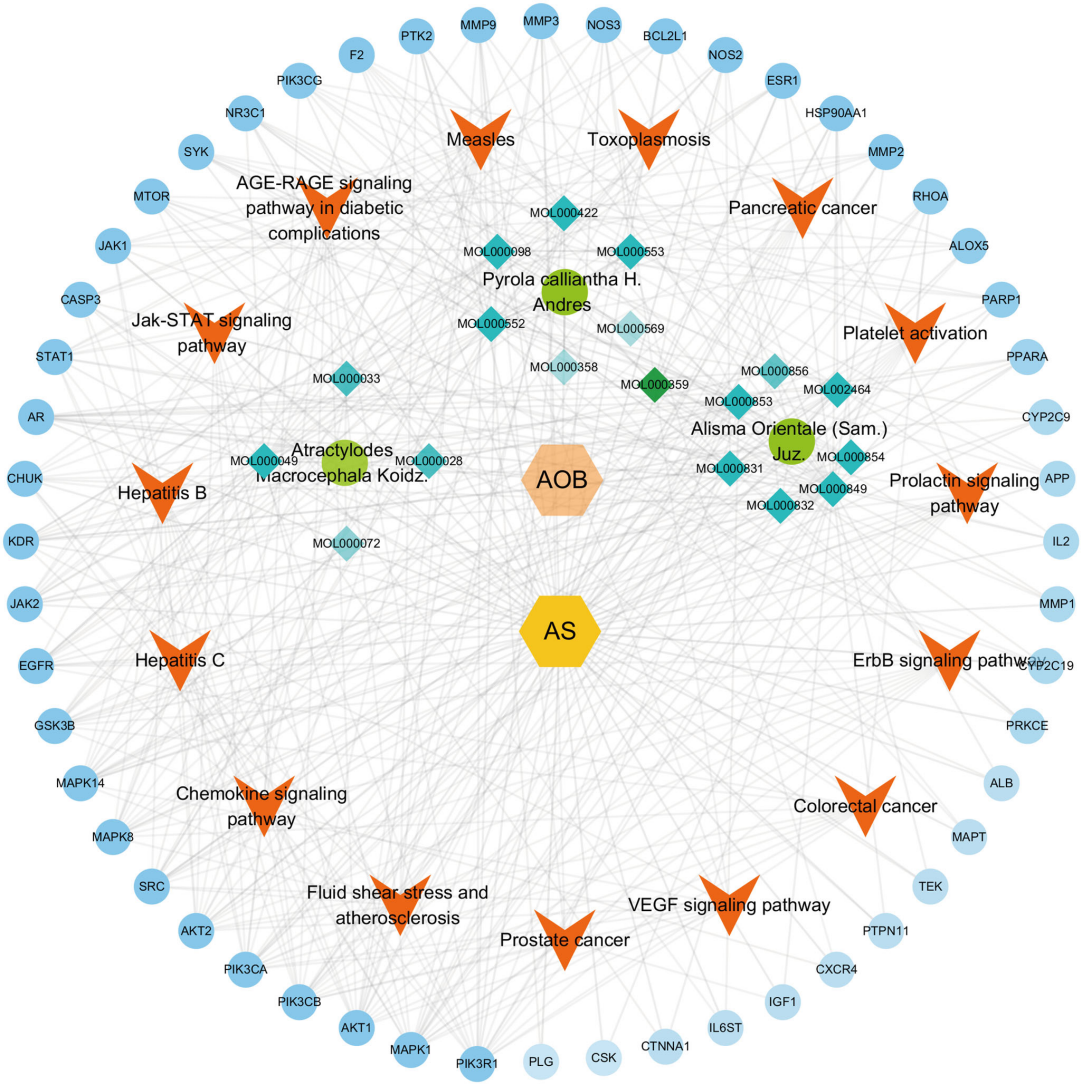
Compound-Disease-Pathway-Target Network. The orange hexagons represent AOB, the yellow hexagons represent AS, the green circles represent three traditional Chinese medicines, the blue diamonds around the green circles are the main components of the medicine, the red arrows represent the pathways, and the outermost blue circles are the targets. The darker the color, the more important the node is.

Combined with the above analysis results, the connection between traditional Chinese medicine, disease, pathways and targets was established. CytoScape3.8.2 was used to construct a Compound-Disease-Pathway-Target network (Figure 5). With the use of the built-in NetworkAnalyzer of CytoScape3.8.2, the network topology parameters of AOB treatment AS were analyzed, and the core components and core role targets were obtained. According to network analysis, 3 main components in the AOB treatment of AS: 16β-methoxyalisol B monoacetate (degree = 36), 3β-acetoxyatractylone (degree = 23), and 5, 2′-dihydroxy-6,7,8-trimethoxyflavone (degree = 19) (Table 8). Therefore, these compounds were regarded as the potential bioactive compounds of AOB against AS. Then, the top 10 core targets were selected according to the comprehensive ranking of degree, closeness and betweenness (Table 9). Interestingly, according to the network analysis results, the PIK3 family and AKT are the core targets of AOB in the treatment of AS, which is consistent with the previous PPI analysis results.

**Table 8.** Main components in the AOB treatment of AS.

Herb name	Mol ID	Betweenness centrality	Closeness centrality	Degree
*Alisma orientale* (Sam.) Juz.	MOL000849	0.120442743	0.539393939	36
*Atractylodes macrocephala* Koidz.	MOL000049	0.056925762	0.465968586	23
*Pyrola calliantha* H. Andres	MOL000552	0.039327035	0.447236181	19

**Table 9.** The top 10 targets of the Compound-Disease-Pathway-Target Network.

Target	Betweenness centrality	Closeness centrality	Degree
PIK3R1	0.025841461	0.497206704	18
MAPK1	0.031190839	0.486338798	18
PIK3CA	0.018435115	0.475935829	17
PIK3CB	0.018435115	0.475935829	17
AKT1	0.020792845	0.486338798	17
AKT2	0.015704295	0.470899471	16
MAPK14	0.035347078	0.470899471	15
MAPK8	0.016521855	0.470899471	15
SRC	0.023938519	0.486338798	15
GSK3B	0.022214607	0.481081081	14

### AOB reversed the aortic plaque area of as in APOE^−/−^ mice stimulated with a high-fat diet

**Figure 6.**
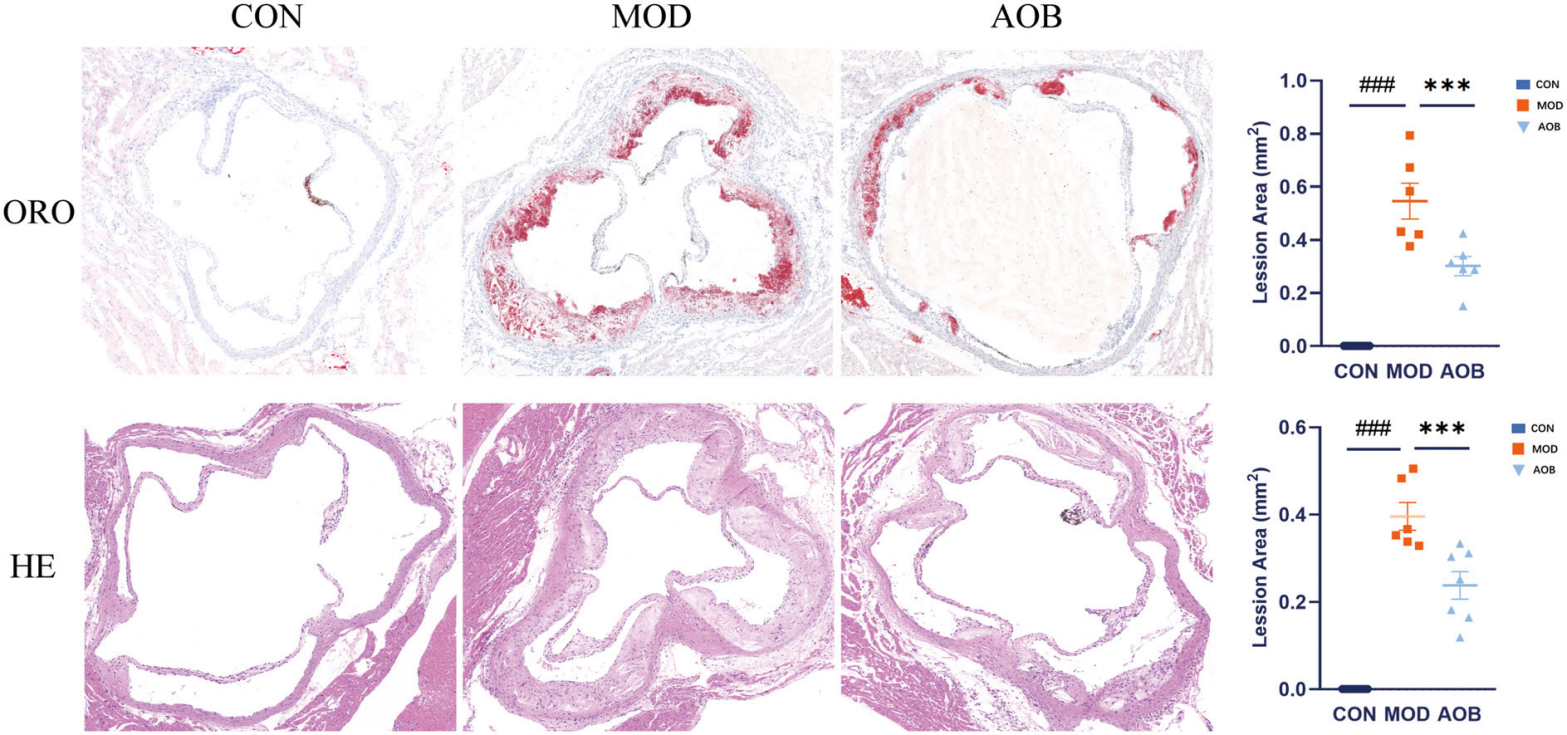
AOB reversed aortic plaque area of AS in APOE^−/−^ mice. HE and ORO stained sections of aortic valve area in the control group, the model group and the AOB group. The atherosclerotic lesion area was quantitatively analyzed by Image J. Data show mean ± SEM values of 6 or more independent samples. ^#^ Represents comparison with the control group, ^###^represents *p* < 0.001; * represents comparison with the model group, *** represents *p* < 0.001.

We performed an AS model with a high-fat diet (HFD). We found that C57BL/6J mice fed with a normal diet for 8 weeks showed no change in arterial plaque area and APOE^−/−^ mice fed with HFD for 8 weeks showed a significant increase in arterial plaque area, which was compared with C57BL/6J mice fed with normal diet (Figure 5). However, after AOB treatment for 8 weeks, the arterial plaque area was significantly reversed both in ORO and HE staining (Figure 6; One-way ANOVA, ORO, F (2, 26) = 62.35, *p* < 0.001; HE, F (2, 26) = 86.91, *p* < 0.001). These data suggested that AOB had a potential effect to treat AS.

### AOB improved four indicators of blood lipids of as in APOE^−/−^ mice stimulated with a high-fat diet

**Figure 7.**
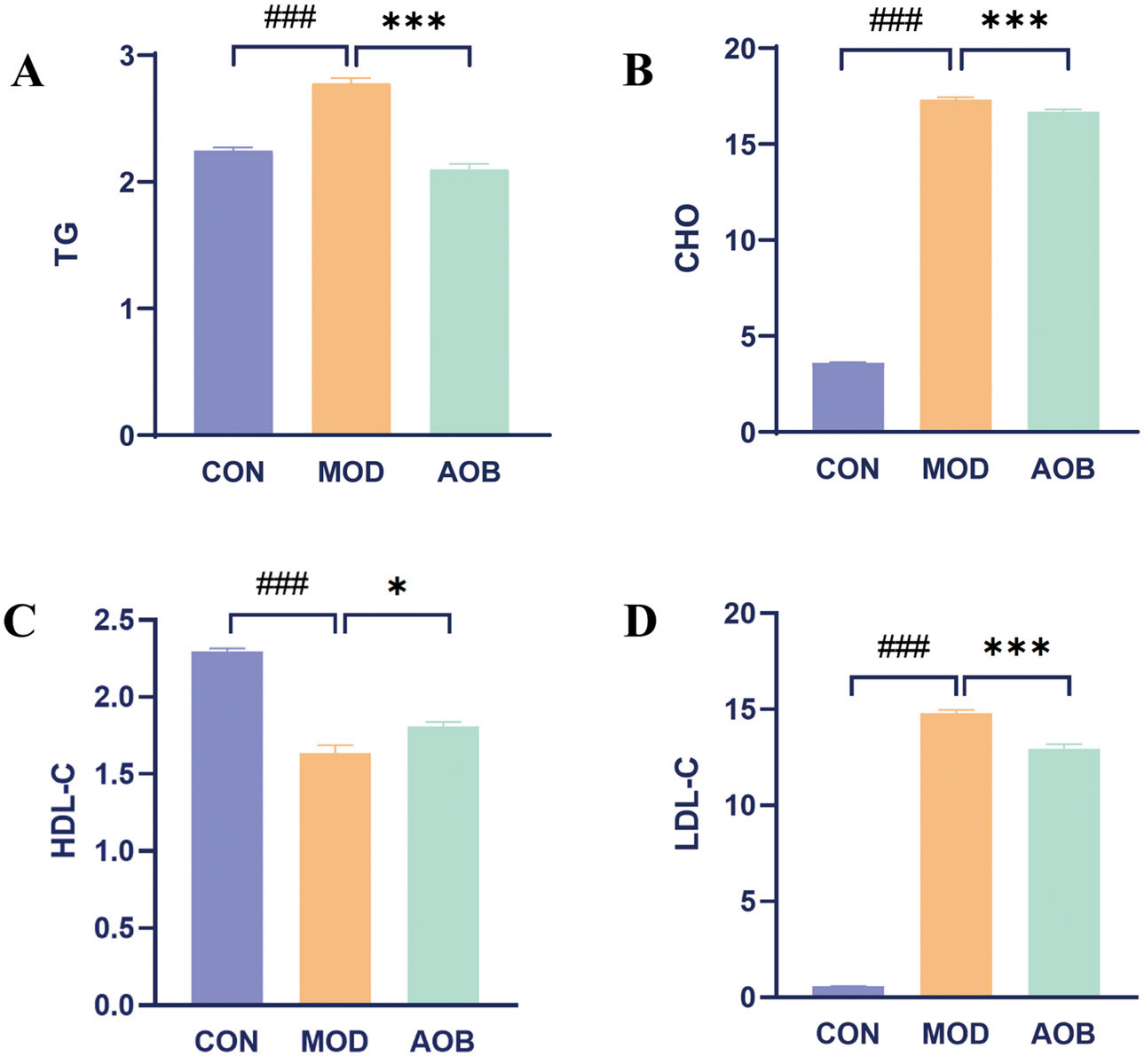
AOB improved four indicators of blood lipid of AS in APOE^−/−^ mice stimulated with high-fat diet. Data show mean ± SEM values of 6 independent samples. # Represents comparison with the control group, ^###^ represents *p* < 0.001; * represents comparison with the model group, * represents *p* < 0.05, *** represents *p* < 0.001.

We then detected four indicators of blood lipids related to AS including TG, CHO, HDL and LDL. We found that TG, CHO, LDL were all increased after HFD feeding in APOE^−/−^ mice for 8 weeks, which were all reversed obviously by AOB for 8 weeks [Figure 7, One-way ANOVA, TG, F (2, 17) = 85.17, *p* < 0.001; CHO, F (2, 17) = 59.30, *p* < 0.001; LDL, F (2, 17) = 19.20, *p* < 0.001]. Moreover, HDL in blood serum was decreased after HFD feeding in APOE^−/−^ mice for 8 weeks, which was also reversed obviously by AOB for 8 weeks [Figure 7, One-way ANOVA, HDL, F (2, 17) = 76.44, *p* < 0.001].

### AOB alleviated as by regulating PI3K pathway

**Figure 8.**
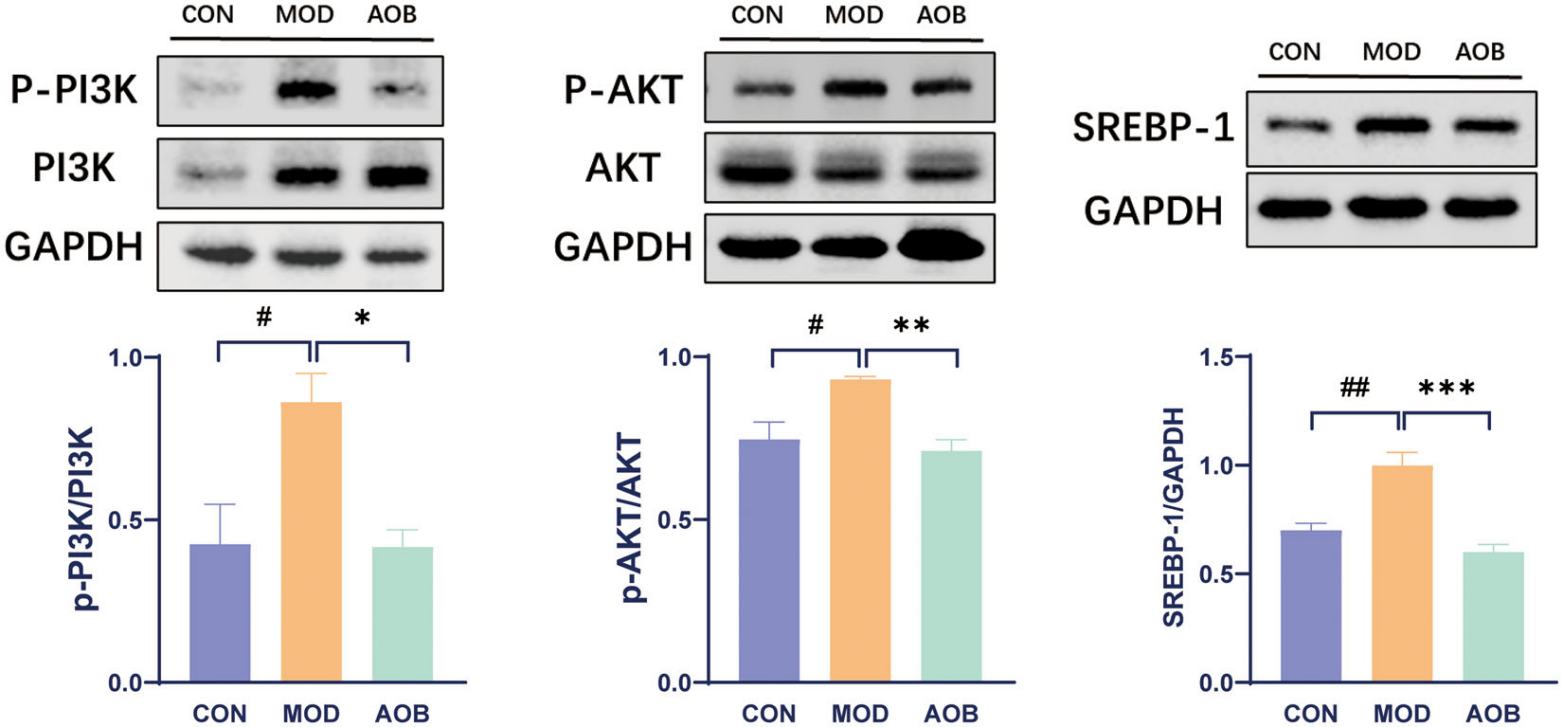
AOB alleviated AS by regulating the PI3K pathway. The expression levels of the PI3K/AKT/SERBP-1 pathway proteins in each group were detected by western blots. The densitometric values of bands were quantitatively analyzed by Image J Densitometric values normalized to those in the model group and are presented as relative intensity. Data show mean ± SEM values of 6 independent samples. ^#^ Represents comparison with the control group, ^#^ represents *p* < 0.05, ^##^ represents *p* < 0.01; * represents comparison with the model group, * represents *p* < 0.05, ** represents *p* < 0.01 *** represents *p* < 0.001.

**Figure 9.**
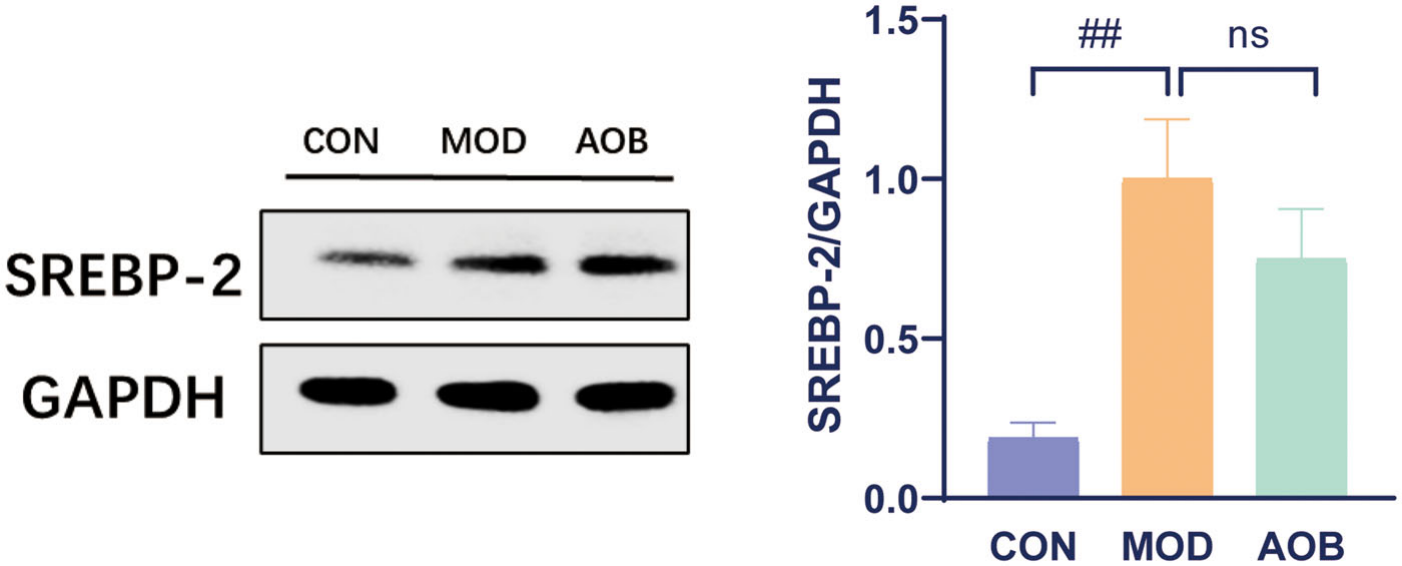
The expression of SREBP-2 in liver after AOB treatment for 8 weeks. The densitometric values of bands were quantitatively analyzed by Image J Densitometric values normalized to those in the model group and are presented as relative intensity. Data show mean ± SEM values of 6 independent samples. ^#^ Represents comparison with the control group, ^##^ represents *p* < 0.01.

Based on our network pharmacological analysis, we chose the PI3K pathway to reveal the underlying mechanism of AOB treatment for AS. Then we measured the levels of PI3K/AKT/SREBP-1 in the liver. The results showed that HFD increased phosphorylated expressions of PI3K/AKT and expression of SREBP-1 in APOE^−/−^ mice compared with C57BL/6J mice fed with a normal diet. Interestingly, AOB treatment for 8 weeks reversed all of them in the liver [Figure 8, One-way ANOVA, pPI3K/PI3K, F (2, 17) = 6.997, *p* = 0.0147; pAKT/AKT, F (2, 17) = 7.925, *p* = 0.0087; SREBP-1, F (2, 17) = 18.14, *p* < 0.001]. Although the expression of SREBP-2 in the liver was significantly increased by HFD, which was not altered after AOB treatment for 8 weeks [Figure 9, One-way ANOVA, F (2, 8) = 9.281 *p* = 0.0082].

## Discussion

The relationship between the lipid metabolism pathway of AOB and AS was still unclear. In this study, we used network pharmacology combining with experiments to reveal the role of AOB in lipid metabolism, which played a major role in the treatment for AS. We found 3 main components in the AOB treatment of AS: 16β-methoxyalisol B monoacetate (degree = 36), 3β-acetoxyatractylone (degree = 23), and 5, 2′-dihydroxy-6,7,8-trimethoxyflavone (degree = 19) according to network analysis. Moreover, core targets were obtained: PIK3R1, AKT1, PIK3CA, MAPK1, PTPN11, EGFR and MAPK4, which might participate in the treatment of AS. In addition, the top 10 significantly enriched (*p < 0.01*) terms in BP, CC and MF of GO analysis were selected and the top 20 pathways with significantly enriched were selected, including the PI3K/AKT/SREBP-1 pathway. Based on the results, we used experiments to identified the therapeutic actions of AOB in AS *via* the PI3K/AKT/SREBP-1 pathway. The results showed that AOB reversed the aortic plaque area of AS, and improved main indicators of blood lipids related to AS and alleviated AS by regulating PI3K/AKT/SREBP-1 pathway in APOE^−/−^ mice stimulated with HFD. Taken together, we firstly demonstrated that AOB was capable of ameliorating AS by regulating the PI3K/AKT/SREBP-1 pathway.

Although a previous study has identified the effects of AOB on the treatment for AS (Zhu, Zhai, et al. 2020), the underlying molecular mechanism was still unclear. The aortic plaque was significantly increased in AS-related diseases and had been shown to be closely related to high-fat diets (HFD) (Pan et al. 2022). In our study, we found a significant increase in the aortic plaque area in APOE^−/−^ mice fed with HFD for 8 weeks, which was reversed by AOB after 8 weeks of continuous treatment. In addition, abnormal blood lipid indicators including TG, CHO, HDL and LDL, which contributed to AS (Jaquish et al. 1996; Barboza et al. 2016), were all relieved by AOB. To sum up, AOB has the effect of alleviating AS in APOE^−/−^ mice stimulated with HFD.

Network pharmacological analysis showed that 3 main components, 16β-methoxyalisol B monoacetate, 3β-acetoxyatractylone, and 5,2′-dihydroxy-6,7,8-trimethoxyflavone, are associated with AOB treatment of AS. 16β-Methoxyalisol B monoacetate from *Alismatis rhizoma* has been identified to have an antibacterial effect (Jin et al. 2012) and the pathophysiology of bacterial is associated with the development of inflammation (Ge et al. 2022; Keir and Chalmers 2022), risk factors for atherosclerosis. Meanwhile, the component was proved to have an inhibitory effect on phosphorylation of the PI3K/Akt pathway (Xu, Zhao, et al. 2009). Moreover, 3β-acetoxyatractylone from *Atractylodis macrocephalae rhizoma* has been indicated to have treatment-related effects of AS (Chen et al. 2017; Li et al. 2018). In addition, 5,2′-dihydroxy-6,7,8-trimethoxyflavone from *Pyrolae herba*, a natural flavonoid, plays a role in lipid decreasing (Lin et al. 2022) and the progression of treatment of AS (Kimura et al. 2022; Liu et al. 2022), which also participate in anti-inflammation (Huang et al. 2022; Liu et al. 2022). Network pharmacological results were further identified in our experimental studies, which showed that AOB was capable of releiving AS by regulating the PI3K/AKT/SREBP-1 pathway.

Hypercholesterolemia is recognized as a major contributor to AS, and lowering blood cholesterol levels is an important means in the treatment of AS. Sterol-regulating element-binding proteins (SREBPs) are a family of transcription factors involved in the biosynthesis of cholesterol, fatty acids, and triglycerides (Moslehi and Hamidi-Zad 2018), consisting of SREBP-1 and SREBP-2. SREBP-1 is responsible for the synthesis of fatty acids and cholesterol, while SREBP-2 only regulates the synthesis of cholesterol (Jeon and Osborne 2012). Studies showed that SREBPs in the liver plays a catalytic role in AS by increasing lipid synthesis (Karasawa et al. 2011; Pérez-Belmonte et al. 2017), and inhibiting SREBP-1 led to lower serum cholesterol levels, further alleviating AS (Karasawa et al. 2011). PI3K/AKT is the upstream signaling pathway of SREBP-1, whose activation increased the expression of SREBP-1 (Jeon and Osborne 2012). Recent studies showed that PI3K/AKT signaling is significantly upregulated in patients with nonalcoholic fatty liver disease, one of the risk factors for AS, and inhibitors against PI3K and AKT have potential regulatory effects on lipid metabolism (Aljabban et al. 2022). Our results showed that phosphorylation of the PI3K/AKT signaling pathway in the liver of AS model mice is significantly activated, resulting in elevated SREBPs. After 8 weeks of AOB administration, phosphorylation of the PI3K/AKT signaling pathway in the liver is restored, further lowering SREBP-1 signaling in the liver instead of SREBP-2. These results indicate that AOB regulated the PI3K/AKT/SERBP-1 pathway leading to therapeutic actions in AS by reducing lipid levels.

## Conclusions

AOB has the therapeutic response of AS, which requires suppression of the PI3K/AKT/SERBP-1 pathway. These were our first findings on AOB’s treatment of AS and the underlying mechanism were associated with inhibition of the PI3K/AKT/SERBP-1 pathway, which suggests that traditional Chinese medicine has an obvious curative effect in the treatment of AS, and had a similar molecular mechanism as other western medicines (Mahtta et al. 2022), which provides strong evidence for our later development and extensive use of traditional Chinese medicine.

## Data Availability

The raw data supporting the conclusions of this manuscript will be available from the corresponding author on reasonable request.
